# Factors affecting morbidity and mortality in traumatic colorectal injuries and reliability and validity of trauma scoring systems

**DOI:** 10.1186/s13017-015-0014-9

**Published:** 2015-05-12

**Authors:** Nurettin Ay, Vahhaç Alp, İbrahim Aliosmanoğlu, Utkan Sevük, Şafak Kaya, Bülent Dinç

**Affiliations:** Diyarbakır Gazi Yaşargil Education and Research Hospital, Transplantation Center, Diyarbakır, Turkey; Department of General Surgery, Diyarbakir Gazi Yaşargil Education and Research Hospital, Diyarbakır, Turkey; Department of General Surgery, Akdeniz University Hospital, Antalya, Turkey; Department of Cardiovascular Surgery, Diyarbakır Gazi Yaşargil Education and Research Hospital, Diyarbakır, Turkey; Department of İnfectious Disease, Diyarbakır Gazi Yaşargil Education and Research Hospital, Diyarbakır, Turkey; Atatürk State Hospital, Antalya, Turkey

**Keywords:** Trauma, Colorectal, TRISS, RTS, ISS

## Abstract

**Background and aim:**

This study aims to determine the factors that affect morbidity and mortality in colon and rectum injuries related with trauma, the use of trauma scoring systems in predicting mortality and morbidity.

**Patients and methods:**

Besides patient demographic characteristics, the mechanism of injury, the time between injury and surgery, accompanying body injuries, admittance Glasgow coma scale (GCS), findings at surgery and treatment methods were also recorded. With the obtained data, the abbreviated injury scale (AIS), injury severity score (ISS), revised trauma score (RTS) and trauma-ISS (TRISS) scores of each patient were calculated by using the 2008 revised AIS.

**Results:**

Of the patients, 172 (88.7 %) were male, 22 (11.3 %) were female and the mean age was 29.15 ± 12.392 (15–89) years. The morbidity of our patients were 32 % and mortality were 12.4 %. ISS (*p* < 0.001), RTS (*p* < 0.001), and the TRISS (*p* < 0.001) on mortality were found to be significant. TRISS (*p* = 0.008), the ISS (*p* < 0.001), the RTS (*p* = 0.03), the trauma surgery interval (TSI, *p* < 0.001) were observed to have significant effects on morbidity.

Regression analysis showed that the ISS (OR 1.1; CI 95 % 1.01–1.2; *p* = 0.02), the RTS (OR 0.37; CI 95 % 0.21–0.67; *p* = 0.001) had significant effects on mortality. While the effects of TSI (OR 5.3; CI 95 % 1.5–18.8; *p* = 0.01) on morbidity were found to be significant.

**Conclusion:**

Predicting mortality by using scoring systems and close postoperative follow up of patients in the risk group may ensure decreases in the rates of morbidity and mortality.

## Introduction

Colorectal injuries are rare in trauma patients and are associated with increased mortality. These injuries constitute 1 % of all trauma patients [1]. Most colonic and rectal injuries occur following penetrating trauma and injury from blunt trauma is uncommon [2]. The mortality associated with colonic trauma has decreased considerably over the last half century; from 40 % during World War II to 1–3 % over the last several decades [3]. Common postoperative complications include systemic complications such as pneumonia, sepsis and complications specific to abdominal surgery such as surgical site infection, intraabdominal abscess, and abdominal sepsis [4].

Staging according to the severity of injury is necessary for the management of trauma and as well as a basic requirement for clinical trials [5]. Trauma scoring systems try to translate the severity of injury into a number. The scores enable physicians to translate different severity of injuries into a common language [6]. Quantitative characterizations of injury are essential for research and meaningful evaluation of patient outcome, quality improvement, and prevention programs [6, 7]. For this purpose, many anatomical and physiological scoring systems are created [8]. There are around 50 scoring systems published for the classification of trauma patients [6]. Some of these scoring systems are new injury severity score (NISS), AIS, ISS, GCS, RTS and TRISS [9]. This study aims to determine the factors that affect morbidity and mortality in colon and rectum injuries related to trauma, to utilize trauma scoring systems for predicting mortality and morbidity.

## Materials and methods

Between January 2005 and December 2010, all the patients who were operated on for blunt or penetrating abdominal injury at Dicle University Faculty of Medicine Hospital were evaluated retrospectively. One ninety four patients with colorectal injury were included in the study.

After the initial evaluation of the patients in the emergency room, a nasogastric catheter, a central venous pressure catheter and a foley catheter was placed into the patients. All the patients were given fluid-electrolyte resuscitation. In the preoperative period, the patients were simultaneously administered two antibiotics (intravenous ceftriaxone 1 *g* and metronidazole 500 mg). Antibiotic therapy was continued in the postoperative period and was stopped on the fifth day. Hemodynamically unstable patients were operated on under emergency conditions. Patients who were hemodynamically stable were assessed by physical examination, laboratory tests and imaging methods (radiography, ultrasonography, computed tomography), peritoneal lavage before proceeding with the surgery. One of these procedures for patients who were scheduled for surgery were performed primary repair+proximal diverting stoma (PDS), resection+anastomosis, primary closure or resection of injured bowel+PDS. Distal washout and presacral drainage were not performed for rectal injuries. Midline laparotomy was performed all patients.

Besides the demographic characteristics of the patients such as age and gender; the mechanism of the injury, the time between injury and surgery, accompanying body injuries (head and neck, face, chest, abdomen, extremities and external structures), vital signs at emergency admittance, GCS at admission, findings during the performed surgery and the treatment methods were also recorded. Follow up data after surgery, observed complications and the duration of time until discharge were evaluated. Trauma and/or operation related complications were defined as morbidity and deaths due to trauma and/or operation were defined as mortality.

With the obtained data, the AIS scores of each patient were calculated by using the 2008 revised AIS (AIS was calculated according to update 2008 dictionary in the www.aaam.org website). The ISS scores were calculated for each patient with the data from the three regions (regions: head & neck, face, chest, abdomen, extremity, external) with the highest scores The RTS were calculated for each patient by using the findings of respiratory rate at admittance to the emergency room, systolic blood pressure and GCS data. The TRISS were calculated for each patient by using the patient ISS, RTS, age and trauma mechanism data. In order to calculate the ISS, RTS and the TRISS, the calculation tool available on the web site www.trauma.org were used.

All colonic injuries were divided into two categories as non-destructive (Flint scale grade 1–2) and destructive (Flint scale grade 3). The patients were divided into two groups, blunt injury group (BIG) and penetrating injury group (PIG), and a comparison was made between these two forms of trauma. The present study was approved by the Dicle University ethics committee and complies with the requirements of the Declaration of Helsinki.

### Statistical analysis

Statistical analysis was performed by using the SPSS for Windows 11.5 (SPSS Inc. Chicago, IL, USA) program. Descriptive statistics were used for the evaluation of the data. The Kolmogorov-Smirnov test was used to determine the distribution of the data. The Chi-square test was used for the comparison of qualitative data between groups and the Mann–Whitney *U* test was used for the comparison of quantitative data. The data were evaluated in terms of mean standard deviation. The regression test was used to determine the factors effecting morbidity and mortality. A *p* value <0.05 was accepted as being statistically significant.

## Results

**Fig. 1:**
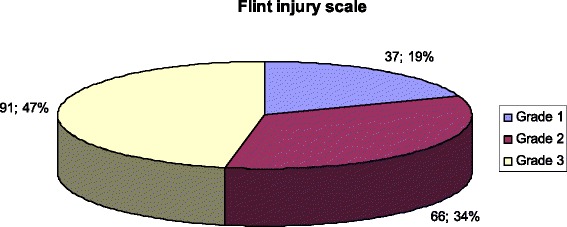
Flint injury scale

**Fig. 2:**
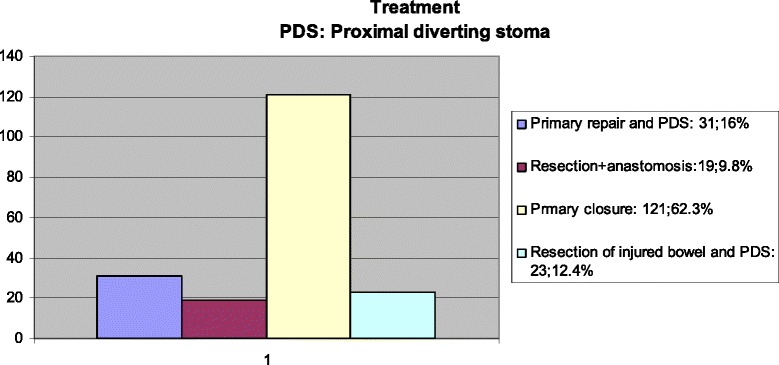
Treatment methods

**Fig. 3:**
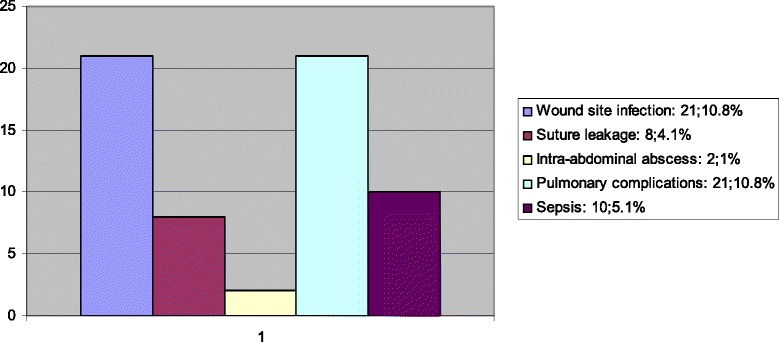
Morbidity after surgery

Between January 2005 and December 2010, 3857 trauma patients were followed and treated by the general surgery department and the incidence of colorectal injury was detected to be 4 %. Colorectal injuries due to blunt trauma comprised 7.7 % of all colorectal injuries. The common causes of injury were gunshot wound (*n* = 128, 66 %), cuts and puncture wounds (*n* = 51, 26.3 %), traffic accidents (*n* = 12, 6.2 %) and falls from a height (*n* = 3, 1.5 %). The majority of the patients with colorectal injuries had Flint grade 1 injuries (53.1 %). Sixty two percent of the cases were managed by primary closure. The morbidity of the patients were 32 % and mortality was 12.4 %. The descriptive data of the study is presented in Figs. 1, 2 and 3.

A hundred and fifty one (77.8 %) patients had accompanying additional organ injuries. Fifty-eight (29.9 %) patients had two or more additional organ injuries. The most common organ injuries seen with colon injuries were small bowel injuries (Table 1).

**Table 1:** The other organs with injuries

Addition organ wounds	Number	Percent
Small bowel	101	52
Stomach	28	14,4
Kidney	22	11,3
Vascular	18	9,2
Diaphragm	17	8,7
Liver	16	8,2
Lung	14	7,2
Spleen	11	5,6
Bladder	8	4,1
Pancreas	3	1,5
Cardiac	3	1,5
Gall bladder	2	1
Ureter	2	1
Urethra	2	1
Oesophagus	1	0,5
Testicle	1	0,5
Multiple small bowel	41	21,1

Comparison of characteristics of BIG and PIG are presented in Table 2. When compared to the PIG, the rates of morbidity (*p* = 0.02), mortality (*p* = 0.02) and the rates of patients treated after 8 hours (*p* = 0.008) of the injury in the BIG were significantly higher. In the BIG, the mean RTS was significantly lower than in the PIG (*p* = 0.037) (Table 2).

**Table 2:** Comparison of characteristics of blunt injury group and penetrating injury group

	PIG (*n* = 179)	BIG (*n* = 15)	*P* value
Age (years), mean ± SD	28.9 ± 12.5	31.2 ± 11.2	0.3
Gender, males, *n* (%)	159 (88.8)	13 (86.6)	0.68
Treatment, *n* (%)			
Primary repair	132 (73.7)	8 (53.3)	0.13
Stoma procedure	47 (26.3)	7 (46.7)	0.13
Flint injury scale			
Grade 1 and 2	96 (53.6)	7 (46.7)	0.8
Grade 3	83 (46.4)	8 (53.3)	0.8
TSI, n(%)			
< 8 hours	165 (92.1)	10 (66.6)	0.008
> 8 hours	14 (7.9)	5 (33.4)	0.008
ISS, mean ± SD	29.7 ± 14.2	33.5 ± 11.8	0.08
RTS, mean ± SD	7.2 ± 1.3	7 ± 0.9	0.03
TRISS, mean ± SD	85 ± 26.1	88.2 ± 12.3	0.07
Hospitalization (days), mean ± SD	11.2 ± 9.3	9.8 ± 8.1	0.23
Morbidity, *n* (%)	53 (29.6)	9 (0.6)	0.02
Wound site infection	18 (10.1)	3 (20)	
Suture leakage	8 (4.5)	-	
Intraabdominal abscess	2 (1.1)	-	
Pulmonary complications	16 (8.9)	5 (33.3)	
Sepsis	9 (5)	1 (6.7)	
Mortality, n (%)	19 (10.6)	5 (33.4)	0.02

*PIG* penetrating injury group, *BIG* blunt injury group, *TSI*, trauma-surgery interval, *ISS* injury severity score, *RTS* revised trauma score, *TRISS* trauma-injury severity score

The effects of gender (*p* = 0.03), the type of trauma (*p* = 0.02), the degree of colon injury (*p* < 0.001), the treatment methods (*p* < 0.001), the presence of additional organ injury (*p* = 0.02), the ISS (*p* < 0.001), RTS (*p* < 0.001), and the TRISS (*p* < 0.001) were found to be significant on mortality rates. The types of trauma (*p* = 0.02), the TRISS (*p* = 0.008), the ISS (*p* < 0.001), the RTS (*p* = 0.03), the treatment methods (*p* = 0.001), the TSI (*p* < 0.001) and the degree of colon injury (*p* < 0.001) were observed to have significant effects on morbidity (Table 3).

**Table 3:** Factors affecting morbidity and mortality following colorectal injury

	Morbidity			Mortality		
	Yes (*n* = 62)	No (*n* = 132)	*P* value	Yes (*n* = 24)	No (*n* = 170)	*P* value
Age, mean ± SD	28.3 ± 11.5	29.5 ± 12.7	0.54	30.5 ± 14.2	28.9 ± 12.1	0.57
Gender (male), *n* (%)	52 (83.8%)	120 (90.9%)	0.15	18 (75%)	154 (90.5%)	0.03
Treatment, *n* (%)						
Primary repair	35 (56.5%)	105 (79.5%)	0.001	8 (33.3%)	132 (77.6%)	<0.001
Stoma procedure	27 (43.5%)	27 (20.4%)		16 (66.7%)	38 (22.4%)	
Flint injury scale, *n* (%)						
Grade 1 and 2	17 (27.4%)	86 (65.2%)	<0.001	3 (12.5%)	100 (58.8%)	<0.001
Grade 3	45 (72.6%)	46 (34.8%)		21 (87.5%)	70 (41.2%)	
TSI, *n* (%)						
<8 hours	47 (75.8%)	128 (97%)	<0.001	19 (79.2%)	156 (91.8%)	0.07
>8 hours	15 (24.2%)	4 (3%)		5 (20.8%)	14 (8.2%)	
ISS	35.4 ± 14.5	27.5 ± 13.1	<0.001	51.7 ± 8.6	27 ± 11.8	< 0,001
RTS	6.86 ± 1.6	7.36 ± 1.1	0.03	4.84 ± 2	7.53 ± 0.73	< 0,001
TRISS	77.2 ± 31	88.9 ± 21.1	0.008	36.6 ± 28.9	92 ± 15.4	< 0,001
Additional organ injuries, *n* (%)						
None	11 (17.7%)	32 (24.2%)	0.07	1 (4.2%)	42 (24.7%)	0.02
1 organ	28 (45.2%)	65 (49.2%)		11 (45.8%)	82 (48.2%)	
2 or more organs	23 (37.1%)	35 (26.5%)		12 (50%)	46 (27.1%)	
Region, *n* (%)						0.8
Caecum	5 (8%)	7 (5.3%)	0.36	0	12 (7%)	
Right colon	8 (13%)	14 (10.6%)		4 (16.6%)	18 (10.6%)	
Transverse colon	18 (29%)	50 (37.9%)	0.36	9 (37.5%)	59 (34.7%)	
Left colon	12 (19.4%)	15 (11.3%)		4 (16.6%)	23 (13.5%)	
Sigmoid colon	9 (14.5%)	32 (24.2%)		5 (20.8%)	36 (21.2%)	
Intraperitoneal rectum	3 (4.8%)	5 (3.9%)		1 (4.2%)	7 (4.1%)	
Extraperitonral rectum	7 (11.3%)	9 (6.8%)		1 (4.2%)	15 (8.9%)	
Group, *n* (%)						
Penetrating	53 (85.5%)	126 (95.6%)	0.02	19 (79.2%)	160 (94.1%)	0.02
Blunt	9 (14.5%)	6 (4.5%)		5 (20.8%)	10 (5.9%)	

*TSI* trauma-surgery interval, *ISS* injury severity score, *RTS* revised trauma score, *TRISS* trauma-injury severity score

Regression analysis showed that Flint injury degree, TSI and treatment type had no significant effect on mortality. The ISS (OR 1.1; CI 95 % 1.01–1.2; *p* = 0.02), the RTS (OR 0.37; CI 95 % 0.21–0.67; *p* = 0.001) and the types of trauma (penetrating-blunt distinction) (OR 0.5; CI 95 % 0.01–0.39; *p* = 0.004) had significant effects on mortality. While the effects of TSI (OR 5.3; CI 95 % 1.5–18.8; *p* = 0.01) and Flint injury degree (OR 3.2; CI 95 % 1.47–7.23; *p* = 0.004) were found to be significant for morbidity; there were no significant correlation observed between morbidity and the TRISS, RTS, ISS or the types of treatment (Table 4).

**Table 4:** Statistics of morbidity and mortality in multivariate analysis

Factors	*P* value	OR	CI % 95 range		*P* value*	OR*	CI % 95 range*	
Group	0.149	0.367	0.94	1433	0.004	0.500	0.006	0.390
Flint injury scale	0.004	3.261	1.471	7.228	0.346	0.300	0.250	3.668
Treatment	0.382	0.701	0.316	1.556	0.222	0.362	0.710	1.847
TSI	0.01	5.303	1.493	18.835	0.132	4.633	0.629	34.151
RTS	0.776	0.906	0.460	1.785	0.001	0.376	0.211	0.671
ISS	0.507	1.015	0.971	1.062	0.019	1.104	1.017	1.199
TRISS	0.834	1.005	0.960	1.052				

*Statistics of Mortality in Multivariate Analysis. *TSI* trauma-surgery interval, *ISS* injury severity score, *RTS* revised trauma score, *TRISS* trauma- injury severity score

## Discussion

It is difficult to determine the incidence of colorectal traumatic injury. In general war series, it has been reported to be as high as 5–10 %. Recently, in the Iraq war data that evaluated more than 3400 trauma patients, the incidence of colorectal injury was found to be 5.1 %. A recent study of colorectal injuries encountered in Afghanistan and Iraq reveals that 71 % of injuries occurred secondary to penetrating trauma, 23 % were secondary to blast, and 5 % occurred during blunt trauma. In civilian series it is observed to be 1–3 %. This rate is lower in blunt trauma [4, 10–12]. During the peace time, 80–90 % of colon injuries are non-destructive colon injuries. In contrast, in war time, 72 % of colon injuries are destructive injuries [7]. In our study destructive injuries ratios were 46.9 %. These high rates of destructive and penetrating injuries may result from the low intensity war between an armed organisation and government forces in our region between 1985 and 2010, and resulting increase of other types of crimes due to social disorder.

In the study by Ng et al., that evaluated 1367 patients with blunt trauma, they found the incidence of colorectal injury to be 0.1 % [13]. Similarly, Carillo et al. found the incidence of colorectal injury to be 0.5 % following blunt trauma [14]. In a multicenter prospective study conducted with 297 patients in the years after 2000 by Demetriades et al., two-thirds of the destructive injuries requiring resection were treated with primary repair; colon related mortality was found to be significantly lower in the primary repair group (0 % and 4 %, *p* = 0.012) and no difference was observed in terms of colon related complications (22 % and 27 %, *p* = 0.373) [4]. In the study by Miller et al., while 153 patients (73 %) without destructive injuries had primary suturing performed, of the 56 patients with destructive injuries, 40 (19 %) had resection and anastomosis and 16 (7.6 %) had stomas [15]. In our study, 140 patients (71 %) had primary repair performed and 54 patients (29 %) had stomas. Morbidity was found to be significantly higher in the ostomy group than in the primary repair group (primary repair: 25 %, ostomy: 50 %, *p* < 0.001). While 70.4 % of the stoma performed patients had destructive injuries, 37.9 % of the primary repair performed patients had destructive injuries (*p* < 0.001). High morbidity in the stoma group by univariate analysis may be associated with high ratio of destructive injury patients. In civilian injuries, the incidence of anastomotic leakage following primary repair is between 1 and 15 % [16, 17]. In their evaluation of 2964 patients, Curran et al. reported this incidence as being 2.4 % [18]. Demetriades at al. in a multi-center prospective trial involving 19 trauma centers that included 297 patients with destructive colon injuries requiring resection, found that there were no difference between primary repair and stoma in term of anastomotic leakage [4]. Cleary et al., reported hemodynamic instability and shock as risk factors for anastomotic leakage and infective complications [19]. In our study, anastomotic leakage was 4.1 %. There was a significant relationship between anastomotic leakage ratio and more than 8 hours of TSI (*p* < 0.001) and the increase in the degree of injury (*p* = 0.004). We detected the anastomotic leakage ratio to be 3.6 % in the primary repair group and 5.6 % in the stoma group (*p* < 0.05). Although we did not reach a clear conclusion as to why more anastomotic leakage was observed in the stoma group, the significantly low levels of RTS observed in the stoma group might suggest that there was more hemodynamic instability in the stoma performed patient group (7.39 vs. 6.69 and *p* < 0.05).

Pinedo, Çöl and Gümüş et al. did not find any significant relationship between age and gender with morbidity in their studies [20–22]. In our study, there was no significant relationship between age and gender with morbidity.

In many studies, the most common organ injury accompanying colon injuries were reported as being small bowel injuries [21–25]. In the studies by Çöl et al., detected at least one accompanying organ injury in 70 % of the patients [21]. In the study by Adesanya et al., where they investigated penetrating colon injuries, the organ injuries most commonly accompanying colon injuries were found to be the small bowel (73.3 %), the liver (25 %) and the stomach (23.3 %) [25]. In our study, the ratio of accompanying organ injury was 77.8 % and the most common accompanying organ injured was the small bowel. In literature, although the second most common organ injuries accompanying colorectal injury after the small bowel were reported as being the spleen or liver [23, 25]. In our study, the spleen ranked fifth. We observed that stomach, kidney and vascular injuries more commonly accompanied colorectal injuries than spleen injuries. This difference can be explained by the fact that higher percentage (92.3 %) of the patients had penetrating injury and 56 % of the colorectal injuries were in the transverse and sigmoid colon. While there was an increase in mortality when colorectal injury was accompanied by two or more organ injuries, (*p* = 0.021), there was no significant increase in observed morbidity (*p* = 0.07). In our study, there was no significant correlation detected between wound localization and mortality or morbidity (*p* > 0.05).

In a study by Singh et al., where they compared the predictive capacity of scoring systems related to trauma; they observed that the RTS and TRISS were better than ISS in predicting the likelihood of survival. Again in the same study: The RTS ranged from 2.746 to 7.8408. There was a graded increase in mortality with decreasing RTS score. There was a graded increase in mortality with increasing ISS scores [6]. In our study univariate analysis, there were significant relationships observed between ISS increase and both RTS and TRISS decrease with increased morbidity and mortality. While in multivariate analysis there were no relationship between these scoring systems and morbidity. Therewere relationships between ISS, RTS and mortality in multivariate analysis. Increased ISS was associated with increased mortality and increased RTS was associated with decreased mortality. We suggest that this arises from the higher ratios of destructive injuries observed in our patients. The positive effect of a TSI < 8 hours on morbidity had been known since the study of Stone and Fabian, which was confirmed with later studies [2, 4, 22, 26]. In our study, 9.8 % of the patients were taken to surgery after 8 hours and 90.2 % were taken to surgery earlier than 8 hours. In patients taken to surgery after 8 hours, the high morbidity was significant (*p* = 0.010 and OR = 5.303 % CI 1.493–18.835).

When the mortalities occurring in the first 24 hours were excluded from the study, the TSI was observed to have significant effect on mortality (*p* < 0.05).

## Conclusion

Objective criteria are needed for evaluation of trauma patients. Using these scoring systems should be used routinely for follow up and predicting morbidity and mortality. Rapid transfer to the hospital, early diagnosis and treatment of patients with possible traumatic colorectal injury will reduce morbidity and mortality rates. Predicting mortality by using scoring systems and a close follow up of the patients postoperatively may reduce morbidity and mortality rates. Our study shows that there are correlations between trauma scoring indexes and morbidity and mortality. This results imply that there is a need for randomized, prospective controlled studies for adopting these scoring indexes for a better patient treatment and care.
